# Supplementary material for: Presence of low virulence chytrid fungi could protect European amphibians from more deadly strains
Source: Nat Commun. 2020 Oct 26;11:5393. doi: 10.1038/s41467-020-19241-7 (PMC7589487; doi:10.1038/s41467-020-19241-7)

Supplementary info

Supplementary Table 1. ***Batrachochytrium dendrobatidis*** **presence in positive locations in Flanders.** Complete overview of tested locations is presented in Supplementary Figure 1.

Species	total number sampled	Number *Bd* positive	Locaction
Ichthyosaura alpestris	15	2	Raffelberg
Lithobates catesbeianus	26	12	Straalmolen
Pelophylax spp.	30	7	Hoogstraten
Pelophylax spp.	30	3	Neerijse
Pelophylax spp.	30	1	Zonhoven
Salamandra salamandra	90	1	Heilig Geestgoed
Ichthyosaura alpestris	23	3	Duffel
Lithobates catesbeianus	12	3	Veerle
Lithobates catesbeianus	21	5	Olmen
Alytes obstetricans	139	1	Groot-Loon
Ichthyosaura alpestris	1	1	's Gravensvoeren
Pelophylax spp.	41	1	Hasselt
Lithobates catesbeianus	47	17	Arendonk
Pelophylax spp.	36	11	Neerijse
Pelophylax spp.	30	14	Arendonk
Alytes obstetricans	78	4	Neerijse
Alytes obstetricans	108	27	's Gravensvoeren

Supplementary Table 2. *B. dendrobatidis* infection dynamics in midwife toads (*Alytes obstetricans*) larvae. Site number refers to Supplementary Figure 1.

Site nr.	Locality	Number of individuals (number *Bd* positive) 2015	Number of individuals (number *Bd* positive) 2016	Number of individuals (number *Bd* positive) 2017	Number of individuals (number *Bd* positive) 2018
1	Neerijse (1)	10 (4)	30 (0)	7 (0)	31(0)
2	Rijkel (2)	31 (0)	0 (0)	12 (0)	0(0)
3	Groot-Loon (3)	84 (0)	24 (1)	0 (0)	31(1)
4	‘s Gravensvoeren (4)	50 (4)	24 (23)	4 (0)	30(0)
5	Sint-Genesius-Rode (5)	0 (0)	0 (0)	0 (0)	0(0)

Supplementary Table 3. Amount of funnel traps used and amount of alpine newts (*Ichthyosaura alpestris*) sampled per pond in March – June 2019.

Pond	Number of used funnel traps				Number of counted alpine newts			
	March	April	May	June	March	April	May	June
1	10	10	10	10	88	69	13	0
2	10	10	10	-[a]	27	73	0	-
3	10	10	10	10	45	46	23	3
4	-	10	10	10	-	117	40	6
5	-	10	10	10	-	156	15	74
6	10	10	10	-	49	165	161	-
7	10	10	10	10	56	105	44	20
8	-	10	10	10	-	57	3	0
9	-	10	10	10	-	28	21	3
10	-	10	10	10	-	96	15	0
11	-	10	10	10	-	41	12	2
12	-	10	10	10	-	40	5	0
13	-	10	10	-	-	65	6	-
14	-	10	10	10	-	29	16	0
15	-	10	10	10	-	63	11	0
16	20	20	20	20	23	70	44	33
17	20	20	20	20	10	29	72	6
18	20	20	20	20	59	22	5	0
19	20	20	20	20	8	30	14	1
20	10	10	10	10	64	7	20	0
21	10	10	10	10	35	14	11	1
22	20	20	20	-	7	17	-	-
23	20	20	20	20	20	36	5	0
24	20	20	20	20	17	33	29	0
25	20	20	20	20	22	15	16	0
26	20	20	20	20	8	19	1	0

[a] no measurements because the pond was not sampled or dried out.

Supplementary Table 4. Local *B. dendrobatidis* GPL isolates isolated in 2015-2018.

Name	Host Species	Life stage	Year of isolation	Province / municipality
*Bd*BE1	*Alytes obstetricans*	Adult	2015	Huldenberg, Flemish-Brabant, Belgium
*Bd*BE2	*Lithobates catesbeianus*	Larvae	2015	Balen, Antwerp, Belgium
*Bd*BE3	*Ichtyosaura alpestris*	Adult	2015	Tubize, Walloon Brabant, Belgium
*Bd*BE4	*Lithobates catesbeianus*	Larvae	2016	Arendonk, Antwerp, Belgium
*Bd*BE5	*Alytes obstetricans*	Larvae	2016	Voeren, Limburg, Belgium
*Bd*BE6	*Ichtyosaura alpestris*	Adult	2016	Voeren, Limburg, Belgium
*Bd*BE7	*Lithobates catesbeianus*	Adult	2016	Laakdal, Antwerp, Belgium
*Bd*BE8	*Lithobates catesbeianus*	Adult	2016	Balen, Antwerp, Belgium
*Bd*BE9	*Lithobates catesbeianus*	Larvae	2016	Laakdal, Antwerp, Belgium
*Bd*BE10	*Lithobates catesbeianus*	Larvae	2016	Balen, Antwerp, Belgium
*Bd*SP11	*Ichtyosaura alpestris*	Adult	2018	El Montnegre i El Corredor, Catalonia, Spain

Supplementary Table 5. Infection parameters in midwife toads for *Bd*JEL423 and local *Bd*GPL isolates. Source data are provided as a Source Data file.

Isolate	Probability of death [mean (95% prediction interval)]	Survival time (days)[1] [mean (95% prediction interval)]	Mean individual GE load [mean± sd]	Max individual GE load [mean± sd]	Total individual GE load[2] [mean± sd]
*Bd*BE1	0.214 (0.037, 0.657)	n/a	881±1501	5567±10219	10377±16282
*Bd*BE3	0.071 (0.003, 0.632)	226.9 (0, 455.4)	276±188	1280±1096	3857±2635
*Bd*BE4	0.071 (0.003, 0.632)	n/a	97±110	533±551	1338±1555
*Bd*BE5	0.042 (0.002, 0.456)	n/a	26±46	198±411	367±650
*Bd*JEL423	0.950 (0.487, 0.997)	31.4 (20.9, 41.9)	119057±75962	296511±320051	414916±372431

[1] Estimated from parametric survival regression

[2] Sum of the weekly GE loads of each individual throughout infection period

Supplementary Table 6. Virulence gene mean fold changes in mRNA expression profile in spores. The data shows the normalized target gene quantities in spores of *Bd*BE1-10 and *Bd*SP11 (n = 4), relative to spores of *Bd*JEL423 which is considered 1 (n = 4). The results are presented as means + s.e.m. with significant differences compared to the *Bd*JEL423 spores indicated in bold (Kruskal–Wallis analysis, followed by pairwise Mann–Whitney U-tests (two-tailed) with a Benjamini-Hochberg adjusted *P* value < 0.05).Target genes were based on Farrer et al. (2017). N.D. = not detected. Source data are provided as a Source Data file.

	Mean Relative Expression + s.e.m.	BDEG 26151	BDEG 24342	CRN 22492	CRN 25085	CRN 23176	ADM 25206	ADM 27285	ADM 22285	ADM 20379	ADM 23888	CBM18 20255	CBM18 28751	CBM18 26087
*Bd*JEL423	spores	1.03+0.13	1.02+0.11	1.03+0.14	1.06+0.23	1.14+0.35	1.01+0.09	1.03+0.15	1.05+0.17	1.02+0.14	1.02+0.13	1.02+0.08	1.03+0.14	1.08+0.22
*Bd*BE1	spores	0.83+0.07<br>(*P* = 0.755)	0.66+0.04<br>(*P* = 0.160)	0.33+0.02<br>(*P* = 0.053)	**0.33+0.01**<br>(*P* = 0.046)	0.25+0.03<br>(*P* = 0.053)	0.64+0.03<br>(*P* = 0.157)	0.71+0.08<br>(*P* = 0.222)	0.95+0.17<br>(*P* = 0.886)	0.51+0.07<br>(*P* = 0.160)	0.61+0.04<br>(*P* = 0.105)	0.87+0.03<br>(*P*= 0.697)	0.50+0.06<br>(*P* = 0.106)	0.74+0.03<br>(*P* = 0.623)
*Bd*BE2	spores	1.65+0.20<br>(*P* = 0.418)	1.42+0.17<br>(*P* = 0.418)	0.80+0.08<br>(*P* = 0.486)	0.94+0.06<br>(*P* = 0.886)	0.64+0.07<br>(*P* = 0.343)	2.22+0.39<br>(*P* = 0.106)	**3.23+0.75**<br>(*P* = 0.048)	1.75+0.39<br>(*P* = 0.628)	1.54+0.11<br>(*P* = 0.209)	1.65+0.21<br>(*P* = 0.105)	2.27+0.20<br>(*P* = 0.088)	1.87+0.28<br>(*P* = 0.157)	2.07+0.21<br>(*P* = 0.314)
*Bd*BE3	spores	1.35+0.18<br>(*P* = 0.539)	1.21+0.16<br>(*P* = 0.486)	0.41+0.04<br>(*P* = 0.053)	**0.43+0.06**<br>(*P* = 0.046)	0.13+0.01<br>(*P* = 0.053)	1.48+0.33<br>(*P* = 0.472)	**3.89+0.74**<br>(*P* = 0.048)	1.44+0.24<br>(*P* = 0.314)	1.13+0.29<br>(*P* = 0.686)	1.32+0.20<br>(*P* = 0.419)	N.D.	0.44+0.06<br>(*P* = 0.106)	1.37+0.13<br>(*P* = 0.668)
*Bd*BE4	spores	1.21+0.27<br>(*P* = 1.000)	1.27+0.21<br>(*P* = 0.486)	0.50+0.08<br>(*P* = 0.090)	**0.50+0.01**<br>(*P* = 0.046)	0.44+0.08<br>(*P* = 0.090)	1.75+0.38<br>(*P* = 0.367)	1.41+0.24<br>(*P* = 0.343)	1.33+0.25<br>(*P* = 0.764)	1.40+0.17<br>(*P* = 0.314)	1.45+0.14<br>(*P* = 0.105)	1.42+0.24<br>(*P* = 0.886)	1.60+0.30<br>(*P* = 0.275)	1.65+0.43<br>(*P* = 0.668)
*Bd*BE5	spores	1.30+0.22<br>(*P* = 0.755)	0.76+0.23<br>(*P* = 0.486)	0.73+0.08<br>(*P* = 0.220)	0.58+0.08<br>(*P* = 0.157)	0.43+0.10<br>(*P* = 0.125)	1.24+0.62<br>(*P* = 1.000)	**6.28+1.25**<br>(*P* = 0.048)	0.95+0.37<br>(*P* = 0.838)	0.78+0.29<br>(*P* = 0.686)	0.77+0.34<br>(*P* = 0.419)	1.30+0.13<br>(*P* = 0.132)	0.59+0.24<br>(*P* = 0.275)	0.94+0.39<br>(*P* = 0.886)
*Bd*BE6	spores	1.52+0.16<br>(*P* = 0.418)	1.17+0.15<br>(*P* = 0.486)	1.46+0.18<br>(*P* = 0.140)	1.31+0.20<br>(*P* = 0.535)	0.58+0.05<br>(*P* = 0.125)	2.07+0.31<br>(*P* = 0.106)	**2.26+0.17**<br>(*P* = 0.048)	2.05+0.24<br>(*P* = 0.160)	1.24+0.11<br>(*P* = 0.686)	1.56+0.16<br>(*P* = 0.105)	2.01+0.30<br>(*P* = 0.132)	1.25+0.10<br>(*P* = 0.377)	1.86+0.26<br>(*P* = 0.314)
*Bd*BE7	spores	1.70+0.26<br>(*P* = 0.418)	1.52+0.16<br>(*P* = 0.486)	1.48+0.25<br>(*P* = 0.140)	1.45+0.21<br>(*P* = 0.535)	0.66+0.20<br>(*P* = 0.125)	2.67+0.57<br>(*P* = 0.106)	**2.96+0.23**<br>(*P* = 0.048)	2.24+0.24<br>(*P* = 0.160)	1.86+0.18<br>(*P* = 0.686)	1.83+0.20<br>(*P* = 0.105)	1.86+0.23<br>(*P* = 0.132)	1.44+0.15<br>(*P* = 0.377)	1.99+0.16<br>(*P* = 0.314)
*Bd*BE8	spores	0.77+0.16<br>(*P* = 0.539)	0.54+0.10<br>(*P* = 0.160)	0.24+0.01<br>(*P* = 0.053)	**0.26+0.02**<br>(*P* = 0.046)	0.09+0.00<br>(*P* = 0.053)	0.88+0.16<br>(*P* = 0.975)	1.73+0.21<br>(*P* = 0.081)	0.74+0.10<br>(*P* = 0.440)	0.50+0.12<br>(*P* = 0.160)	0.62+0.12<br>(*P* = 0.179)	0.71+0.08<br>(*P* = 0.578)	0.66+0.10<br>(*P* = 0.251)	0.69+0.13<br>(*P* = 0.440)
*Bd*BE9	spores	1.57+0.52<br>(*P* = 0.755)	0.78+0.18<br>(*P* = 0.486)	0.3+0.06<br>(*P* = 0.053)	**0.28+0.05**<br>(*P* = 0.046)	0.10+0.01<br>(*P* = 0.053)	1.08+0.19<br>(*P* = 0.594)	**2.13+0.25**<br>(*P* = 0.048)	1.21+0.26<br>(*P* = 0.838)	0.76+0.15<br>(*P* = 0.686)	0.92+0.14<br>(*P* = 0.886)	0.86+0.18<br>(*P* = 0.519)	1.12+0.30<br>(*P* = 1.000)	0.99+0.33<br>(*P* = 0.886)
*Bd*BE10	spores	1.25+0.18<br>(*P* = 0.539)	0.81+0.08<br>(*P* = 0.486)	0.25+0.01<br>(*P* = 0.053)	**0.24+0.03**<br>(*P* = 0.046)	0.15+0.02<br>(*P* = 0.053)	1.48+0.16<br>(*P* = 0.251)	1.36+0.09<br>(*P* = 0.222)	1.08+0.09<br>(*P* = 0.886)	1.46+0.38<br>(*P* = 0.686)	1.09+0.12<br>(*P* = 0.886)	1.51+0.16<br>(*P* = 0.519)	1.27+0.15<br>(*P* = 0.275)	1.37+0.31<br>(*P* = 0.838)
*Bd*SP11	spores	0.74+0.25<br>(*P* = 0.539)	0.59+0.14<br>(*P* = 0.486)	0.42+0.04<br>(*P* = 0.053)	**0.37+0.03**<br>(*P* = 0.046)	0.24+0.02<br>(*P* = 0.053)	0.71+0.19<br>(*P* = 0.472)	1.06+0.50<br>(*P* = 0.343)	0.56+0.17<br>(*P* = 0.314)	0.56+0.22<br>(*P* = 0.440)	0.46+0.13<br>(*P* = 0.105)	0.81+0.16<br>(*P* = 0.634)	0.31 + 0.11<br>(*P* = 0.106)	0.43 + 0.14<br>(*P* = 0.314)

Supplementary Table 7. Virulence gene mean fold changes in mRNA expression profile in spores and spores + tissue. The data shows the normalized target gene quantities in spores of *BdD*BE1-10, *Bd*SP11 and *Bd*JEL423 when they were incubated with skin tissue of midwife toads (*A. obstetricans*) (n = 4) for 2 h relative to freshly collected spores of the respective isolate (n = 4) which is considered 1. The results are presented as means + s.e.m. with significant differences compared to the respective spore control indicated in bold (Kruskal–Wallis analysis, followed by pairwise Mann–Whitney U-tests (two-tailed), with a *P* value < 0.05). Target genes were based on Farrer et al. (2017). N.D. = not detected. Source data are provided as a Source Data file.

	Mean Relative Expression + s.e.m.	BDEG 26151	BDEG 24342	CRN 22492	CRN 25085	CRN 23176	ADM 25206	ADM 27285	ADM 22285	ADM 20379	ADM 23888	CBM18 20255	CBM18 28751	CBM18 26087
*Bd*JEL423	spores	1.03+0.13	1.02+0.11	1.03+0.14	1.06+0.23	1.14+0.35	1.01+0.09	1.03+0.15	1.05+0.17	1.02+0.14	1.02+0.13	1.02+0.08	1.03+0.14	1.08+0.22
	spores + tissue	1.33+0.08<br>(*P* = 0.114)	1.04+0.07<br>(*P* = 0.686)	**2.11+0.28**<br>(*P* = 0.029)	1.88+0.17<br>(*P* = 0.057)	1.44+0.17<br>(*P* = 0.486)	1.10+0.19<br>(*P* = 1.000)	0.77+0.14<br>(*P* = 0.343)	0.91+0.02<br>(*P* = 1.000)	0.8+0.03<br>(*P* = 0.114)	0.96+0.04<br>(*P* = 0.886)	**1.52+0.11**<br>(*P* = 0.015)	1.10+0.16<br>(*P* = 0.686)	1.09+0.08<br>(*P* = 0.886)
*Bd*BE1	spores	1.01+0.08	1.01+0.07	1.00+0.06	1.00+0.04	1.02+0.12	1.00+0.05	1.02+0.12	1.04+0.18	1.03+0.13	1.01+0.06	1.00+0.04	1.02+0.12	1.00+0.04
	spores + tissue	1.31+0.25<br>(*P* = 0.486)	**1.64+0.20**<br>(*P* = 0.029)	**4.83+0.52**<br>(*P* = 0.029)	**6.08+0.80**<br>(*P* = 0.029)	**3.73+0.71**<br>(*P* = 0.029)	1.63+0.54<br>(*P* = 0.400)	**2.22+0.41**<br>(*P* = 0.029)	0.98+0.19<br>(*P* = 0.686)	1.52+0.10<br>(*P* = 0.057)	1.41+0.22<br>(*P* = 0.200)	**1.97+0.15**<br>(*P* = 0.029)	1.48+0.14<br>(*P* = 0.114)	**1.45+0.11**<br>(*P* = 0.029)
*Bd*BE2	spores	1.02+0.12	1.02+0.12	1.01+0.11	1.01+0.07	1.02+0.11	1.04+0.18	1.07+0.25	1.07+0.24	1.01+0.07	1.02+0.13	1.01+0.09	1.04+0.15	1.01+0.10
	spores + tissue	0.56+0.12<br>(*P* = 0.057)	**0.42+0.07**<br>(*P* = 0.029)	**2.65+0.19**<br>(*P* = 0.029)	**2.45+0.27**<br>(*P* = 0.029)	**2.94+0.52**<br>(*P* = 0.029)	0.46+0.15<br>(*P* = 0.057)	**0.30+0.08**<br>(*P* = 0.029)	0.57+0.12<br>(*P* = 0.300)	**0.39+0.12**<br>(*P* = 0.029)	**0.41+0.08**<br>(*P* = 0.029)	0.49+0.12<br>(*P* = 0.057)	**0.41+0.12**<br>(*P* = 0.029)	**0.42+0.12**<br>(*P* = 0.029)
*Bd*BE3	spores	1.03+0.14	1.03+0.13	1.02+0.11	1.03+0.13	1.01+0.06	1.08+0.24	1.06+0.20	1.04+0.17	1.08+0.28	1.03+0.16	N.D.	1.03+0.13	1.01+0.10
	spores + tissue	**0.44+0.08**<br>(*P* = 0.029)	1.09+0.09<br>(*P* = 0.886)	**2.44+0.36**<br>(*P* = 0.029)	**2.23+0.20**<br>(*P* = 0.029)	**2.71+0.16**<br>(*P* = 0.029)	0.64+0.08<br>(*P* = 0.200)	**0.31+0.01**<br>(*P* = 0.029)	**0.47+0.07**<br>(*P* = 0.029)	**0.43+0.09**<br>(*P* = 0.029)	**0.43+0.03**<br>(*P* = 0.029)	N.D.	**0.43+0.08**<br>(*P* = 0.029)	**0.47+0.11**<br>(*P* = 0.029)
*Bd*BE4	spores	1.09+0.25	1.05+0.17	1.04+0.16	1.00+0.03	1.05+0.20	1.09+0.24	1.05+0.18	1.06+0.20	1.02+0.12	1.02+0.10	1.05+0.18	1.06+0.20	1.12+0.29
	spores + tissue	**0.20+0.04**<br>(*P* = 0.029)	**0.13+0.02**<br>(*P* = 0.029)	**0.35+0.03**<br>(*P* = 0.029)	**0.28+0.05**<br>(*P* = 0.029)	**0.21+0.05**<br>(*P* = 0.029)	**0.09+0.02**<br>(*P* =)	0.83+0.15<br>(*P* = 0.486)	**0.24+0.02**<br>(*P* = 0.029)	**0.06+0.01**<br>(*P* = 0.029)	**0.09+0.02**<br>(*P* = 0.029)	**0.28+0.03**<br>(*P* = 0.029)	**0.09+0.02**<br>(*P* = 0.029)	**0.08+0.01**<br>(*P* = 0.029)
*Bd*BE5	spores	1.05+0.17	1.19+0.36	1.02+0.11	1.02+0.13	1.09+0.25	2.05+1.03	1.06+0.21	1.38+0.53	1.60+0.60	1.65+0.73	1.02+0.10	2.87+1.15	2.11+0.88
	spores + tissue	**0.17+0.01**<br>(*P* = 0.029)	**0.12+0.02**<br>(*P* = 0.029)	**0.16 +0.02**<br>(*P* = 0.029)	**0.20+0.01**<br>(*P* = 0.029)	**0.16+0.01**<br>(*P* = 0.029)	0.19+0.01<br>(*P* = 0.343)	1.36+0.19<br>(*P* = 0.343)	**0.17+0.03**<br>(*P* = 0.029)	0.12+0.03<br>(*P* = 0.114)	0.20+0.03<br>(*P* = 0.200)	**0.31+0.03**<br>(*P* = 0.029)	0.21+0.06<br>(*P* = 0.343)	0.13+0.02<br>(*P* = 0.343)
*Bd*BE6	spores	1.02+ 0.11	1.03+0.13	1.03+0.13	1.04+0.16	1.01+0.09	1.03+0.15	1.01+0.08	1.02+0.12	1.01+0.09	1.02+0.10	1.03+0.15	1.01+0.08	1.04+0.14
	spores + tissue	**0.29+0.03**<br>(*P* = 0.029)	**0.27+0.03**<br>(*P* = 0.029)	1.69+0.32<br>(*P* = 0.114)	1.78+0.26<br>(*P* = 0.114)	1.54+0.28<br>(*P* = 0.343)	**0.23+0.02**<br>(*P* = 0.029)	**0.23+0.02**<br>(*P* = 0.029)	**0.21+0.06**<br>(*P* = 0.029)	**0.27+0.04**<br>(*P* = 0.029)	**0.30+0.06**<br>(*P* = 0.029)	**0.32+0.10**<br>(*P* = 0.029)	**0.20+0.04**<br>(*P* = 0.029)	**0.16+0.06**<br>(*P* = 0.029)
*Bd*BE7	spores	1.04+0.16	1.02+0.11	1.04+0.17	1.03+0.15	1.13+0.34	1.08+0.23	1.01+0.08	1.02+0.11	1.01+0.10	1.02+0.11	1.02+0.13	1.02+0.11	1.01+0.08
	spores + tissue	**0.34+0.08**<br>(*P* = 0.029)	**0.20+0.03**<br>(*P* =0.029)	0.44+0.14<br>(*P* = 0.057)	**0.36+0.08**<br>(*P* = 0.029)	0.52 +0.18<br>(*P* = 0.343)	**0.20+0.02**<br>(*P* = 0.029)	**0.55+0.11**<br>(*P* = 0.029)	**0.23+0.03**<br>(*P* = 0.029)	**0.18+0.03**<br>(*P* = 0.029)	**0.22+0.01**<br>(*P* = 0.029)	**0.38+0.05**<br>(*P* = 0.029)	**0.26+0.05**<br>(*P* = 0.029)	**0.19+0.04**<br>(*P* = 0.029)
*Bd*BE8	spores	1.10+0.23	1.07+0.20	1.00+0.03	1.01+0.09	1.00+0.05	1.06+0.19	1.02+0.12	1.03+0.14	1.14+0.28	1.07+0.21	1.02+0.12	1.04+0.16	1.07+0.20
	spores + tissue	1.14+0.32<br>(*P* = 1.000)	0.7+0.20<br>(*P* = 0.343)	**2.26+0.47**<br>(*P* = 0.029)	2.01+0.39<br>(*P* = 0.057)	**3.23+0.48**<br>(*P* = 0.029)	0.99+0.25<br>(*P* = 0.886)	1.94+0.15<br>(*P* = 0.057)	0.87+0.32<br>(*P* = 0.343)	1.08+0.41<br>(*P* = 0.886)	0.9+0.28<br>(*P* = 0.686)	1.29+0.23<br>(*P* = 0.343)	2.32+1.61<br>(*P* = 1.000)	2.32+1.41<br>(*P* =0.886)
*Bd*BE9	spores	1.17+0.39	1.09+0.25	1.07+0.20	1.06+0.19	1.02+0.12	1.07+0.19	1.02+0.12	1.09+0.23	1.08+0.22	1.04+0.16	1.08+0.23	1.11+0.30	1.15+0.39
	spores + tissue	1.25+0.04<br>(*P* = 0.343)	1.10+0.11<br>(*P* = 1.000)	**8.47+1.71**<br>(*P* = 0.029)	**9.43+2.53**<br>(*P* = 0.029)	**13.7+2.65**<br>(*P* = 0.029)	1.14+0.31<br>(*P* = 0.886)	**0.44+0.06**<br>(*P* = 0.029)	1.06+0.26<br>(*P* = 1.000)	1.14+0.20<br>(*P* = 0.886)	1.10+0.23<br>(*P* = 0.686)	1.86+0.36<br>(*P* = 0.114)	1.21+0.20<br>(*P* = 0.886)	1.38+0.17<br>(*P* = 0.486)
*Bd*BE10	spores	1.03+0.15	1.01+0.10	1.00+0.05	1.02+0.12	1.02+0.11	1.02+0.11	1.01+0.06	1.01+0.08	1.11+0.29	1.02+0.11	1.02+0.11	1.02+0.12	1.09+0.24
	spores + tissue	**0.12+0.02**<br>(*P* = 0.029)	**0.08+0.02**<br>(*P* = 0.029)	**0.15+0.02**<br>(*P* = 0.029)	**0.16+0.01**<br>(*P* = 0.029)	**0.13+0.02**<br>(*P* = 0.029)	**0.07+0.02**<br>(*P* = 0.029)	0.85+0.13<br>(*P* = 0.686)	**0.12+0.02**<br>(*P* = 0.029)	**0.03+0.01**<br>(*P* = 0.029)	**0.07+0.02**<br>(*P* = 0.029)	**0.18+0.02**<br>(*P* = 0.029)	**0.05+0.01**<br>(*P* = 0.029)	**0.06+0.01**<br>(*P* = 0.029)
*Bd*SP11	spores	1.17+0.40	1.07+0.26	1.01+0.09	1.01+0.09	1.01+0.10	1.10+0.29	1.30+0.61	1.11+ 0.33	1.20+0.47	1.11+0.31	1.06+0.21	1.16+0.39	1.13+0.36
	spores + tissue	**3.42+0.28**<br>(*P* = 0.029)	1.50+0.25<br>(*P* = 0.200)	**2.84+0.23**<br>(*P* = 0.029)	**3.03+0.18**<br>(*P* = 0.029)	**5.28+0.73**<br>(*P* = 0.029)	3.09+0.97<br>(*P* = 0.057)	1.55+0.47<br>(*P* = 0.343)	2.75+0.85<br>(*P* = 0.114)	6.30+2.06<br>(*P* = 0.057)	2.33+0.63<br>(*P* = 0.114)	**2.75+0.08**<br>(*P* = 0.029)	2.32+0.59<br>(*P* = 0.200)	1.62+0.45<br>(*P* = 0.486)

Supplementary Table 8. Infection parameters in midwife toads (*A. obstetricans*) exposed to *Bd*JEL423 after infection with local *Bd*GPL isolates. Source data are provided as a Source Data file.

Isolate[1]	Probability of death [mean (95% prediction interval)][2]	Survival time (days)[2] [mean (95% prediction interval)]	Mean individual GE load[2] [mean± sd]	Max individual GE load[2] [mean± sd]	Total individual GE load[3,4] [mean± sd]
*Bd*BE1	0.400 (0.100, 0.799)	113 (34, 192)	1835±2034*	6542±6182*	17194±18706*
*Bd*BE3	0.167 (0.023, 0.631)*	187 (0, 376)*	2141±2271*	8418±7625*	18560±14765*
*Bd*BE4	0.800 (0.309, 0.973)	71 (36, 106)	11166±9414	75976±75422	106426±84111
*Bd*BE5	0.818 (0.493, 0.954)	62 (42, 82)	29813±36194	100180±136009	184770±181753
Control	0.833 (0.369, 0.977)	54 (31, 77)	15180±9258	57667±32762	91718±52306

[1] Control indicates animals without prior *Bd* exposure (control group from initial infection experiment)

[2] Asterisk * indicates significant difference from control group (GLM, α=0.05); when *Bd*BE1-3 and *Bd*BE4-5 were paired, group *Bd*BE1-3 was significantly different from control group.

[3] Estimated from parametric survival regression

[4] Sum of the weekly GE loads of each individual throughout infection period

Supplementary Table 9. Infection parameters in urodelan species exposed to *B. salamandrivrorans* after infection with local *Bd*GPL isolate. Source data are provided as a Source Data file.

Species	Probability of death[1] [mean (95% prediction interval)][2]	Survival time (days)[2,3] [mean (95% prediction interval)]	Mean individual Bsal GE load[2] [mean± sd]	Max individual Bsal GE load[2] [mean± sd]	Total individual Bsal GE load[2,4] [mean± sd]
P. waltl					
Bd only	0.056 (0.003, 0.548)	n/a	n/a	n/a	n/a
Bd-Bsal	0.056 (0.003, 0.548)	n/a	35 ± 25*	225 ± 201*	669 ± 634*
Bsal only	0.389 (0.133, 0.725)	n/a	446 ± 556*	1831 ± 1641*	4006 ± 2818*
S. salamandra					
Bd only	0.050 (0.003, 0.513)	n/a	n/a	n/a	n/a
Bd-Bsal	0.950 (0.487, 0.997)*	38 (30, 46)	402 ± 222	865 ± 448	1170 ± 537
Bsal only	0.950 (0.487, 0.997)*	41 (32, 50)	412 ± 279	726 ± 534	990 ± 569
T. marmoratus					
Bd only	0.071 (0.003, 0.632)	n/a	n/a	n/a	n/a
Bd-Bsal	0.438 (0.149, 0.776)	179 (119, 239)*	651 ± 404	2833 ± 1353	6663 ± 3627
Bsal only	0.938 (0.413, 0.997)*	40 (31, 49)*	16004 ± 38413	75675 ± 193288	77291 ± 193231

[1] Asterisk * indicates significant difference from control group (α=0.05)

[2] Asterisk * indicates significant difference between *Bd-Bsal* and *Bsal*-only groups (α=0.05)

[3] Estimated from parametric survival regression (models for *P. waltl* could not be fit)

[4] Sum of the weekly GE loads of each individual throughout infection period

Supplementary Table 10. List of genes and sequences of the primers used for quantitative PCR analysis.

Gene	Forward primer	Reverse primer	Ref
α-centractin	GCAGCATGGAGTTGTCACTG	AGCTTGGTCACGATTGGAAC	Farrer et al., 2017
APRT	GGTTGCCACTTGGAGTCTGT	ATGGCTGGATGGAAACTCTG	Verbrugghe et al., 2019
TUB	CTCTCGGTGGTGGTACTGGT	AGGGTATTCCTCGCGAATCT	Verbrugghe et al., 2019
Ctsyn1	TCCTCAGCAGCTCCTATTCG	CTCGACGTCCTTTTTCAGGA	Verbrugghe et al., 2019
ADM_20379	CTGGTATGGACGCTCTCGTT	AGACTAAGCCAGTCGCTCCA	Farrer et al., 2017
ADM_23888	CACCCAACGAGTTCAAGGTT	GCCGTCTTGGATATGGACAG	Farrer et al., 2017
ADM_27285	GCAGATGGTCAACCTGGAGT	CCACTGTCTCGATTCGGATT	Farrer et al., 2017
ADM_25206	GGTCTTGACAGCCAAATCGT	CTCTAGCCTCACCGTCGAAC	Farrer et al., 2017
ADM_22285	GGTGTCTCAGGTCGGTTGAC	GATTCCTTGGCAGACACGAT	Farrer et al., 2017
CBM18_20255	ATCTTGCTTGACACCCGAAG	GTGACTTGGCTGATGCCTTT	Farrer et al., 2017
CBM18_28751	CGTGTGGACGTCGATACAAC	CATCCAACACTGCAATGAGC	Farrer et al., 2017
CBM18_26087	TCGTGAACTAACGCAACAGC	CAGACGGTACTTGACGCAGA	Farrer et al., 2017
BDEG_24342	CCGGCTACAAGCTTGTGAGA	GTGTTGGATCCAGGACCCTG	Farrer et al., 2017
BDEG_26151	CAGCTGATGAAGATGGCTCA	GGTTCGTTAGTCGGGACAGA	Farrer et al., 2017
CRN_23176	AAACGCCCTTCGCTTCGATA	TCTTTCTCCAAGCTGAGCGG	Farrer et al., 2017
CRN_25085	CTCCCGGTTCGACATCACAA	GAACAGCGAACCACAGCTTG	Farrer et al., 2017
CRN_22492	CTCCCGGTTCGACATCACAA	GAACAGCGAACCACAGCTTG	Farrer et al., 2017

Supplementary Figure 1. Sampling of amphibian populations for *B. dendrobatidis* screening during 2015-2016. The size of the blue circles represents the number of sampled amphibians. The red colour illustrates the percentage *B. dendrobatidis* positive amphibians (Supplementary Table 1). Numbers refer to site locations in supplementary Table 2. Source data are provided as a Source Data file.

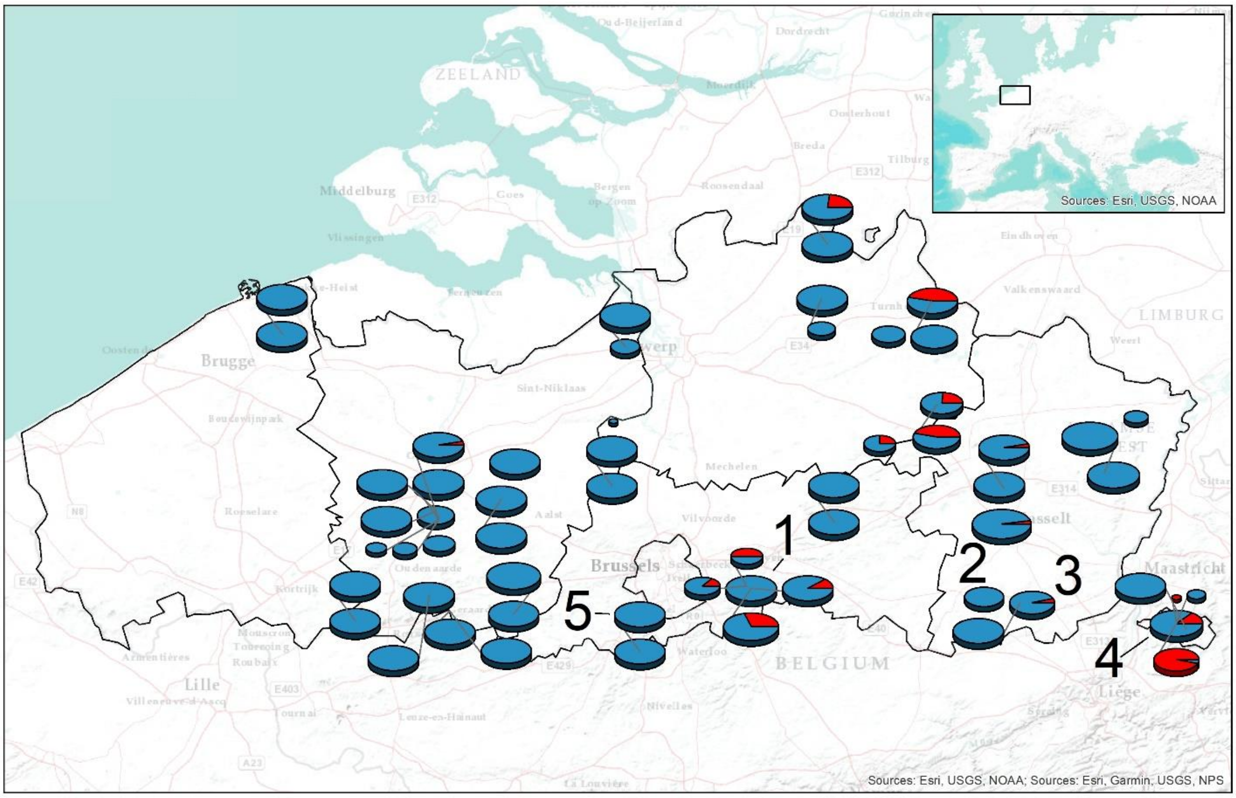

Supplementary Figure 2. Body condition (i.e. Scaled Mass Index) for alpine newt (*I. alpestris*) populations in presence (BD+, n = 41 biologically independent animals**) or absence (BD-**, n = 1728 biologically independent animals**) of *B. dendrobatidis*** presented as boxplots, indicating the median, quartiles, minima and maxima. The violin plot outlines illustrate kernel probability density, i.e. the width of the area represents the proportion of the data located there. Source data are provided as a Source Data file.

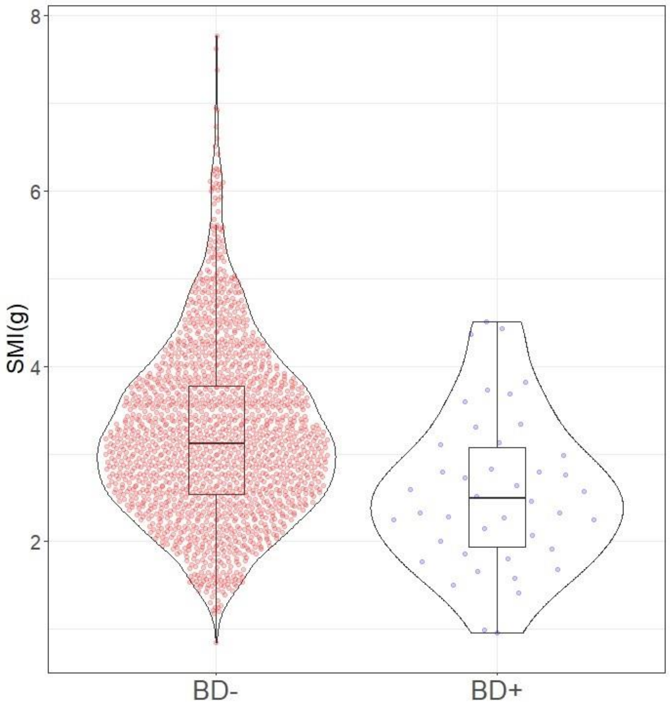

Supplementary Figure 3. Dynamics of fungal load in experimentally infected common toad (*Bufo bufo*), natterjack taod (*Epidalea calamita*), European tree frog (*Hyla arborea*), alpine newt *(I. alpestris*), spadefoot toad *(Pelobates fuscus*), fire salamander *(Salamandra salamandra)* and great crested newt (*Triturus cristatus)*. Fire salamanders (linear model: F(1,5) = 143.1, regression coefficient β = 1.46, two-sided *t*-test on regression coefficient t = 11.96, $p < 0.001$), spadefoot toads (F(1,8) = 26.8, β = 2.69, t = 5.18, $p < 0.001$), European tree frogs (F(1,7) = 21.1, β = 2.21, t = 4.59, p = 0.003) and alpine newts (F(1,6) = 7.71, β = 1.33, t = 2.78, p = 0.03) all showed significantly lower mean infection loads by local *Bd*GPL compared to the virulent *Bd*JEL423. The same differences were observed for maximum and total loads. Source data are provided as a Source Data file.

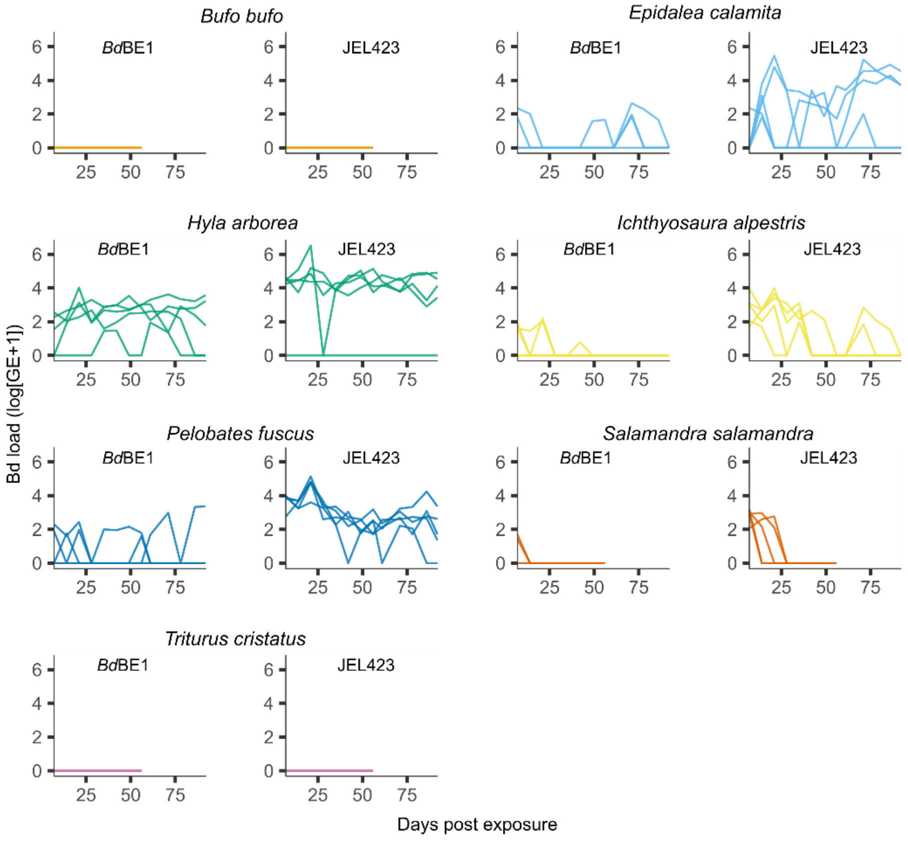

Supplementary Figure 4. **Growth of *Bd* in the mucosome of midwife toads (*A. obstetricans*).** The corrected growth (expressed in GE-Mean (GE NC)) of 5 different *Bd* isolates, *Bd*BE1, *Bd*BE3, *Bd*BE4, *Bd*BE5 and *Bd*JEL423 is shown after a 5 day incubation in the mucosome of midwife toads. The experiment was carried out in triplicate and individual data points are shown (red) with the median ± s.e.m depicted by the horizontal bars. Source data are provided as a Source Data file.

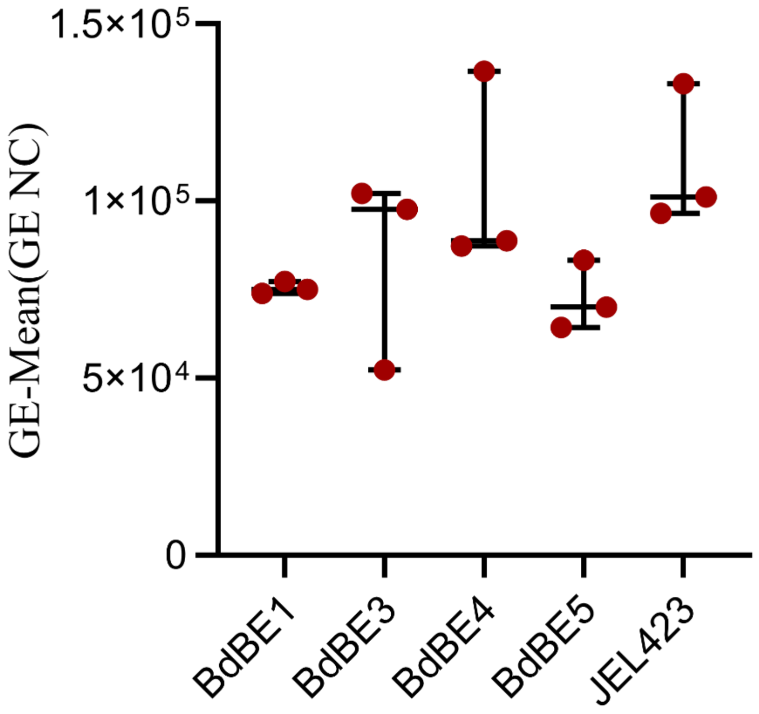

Supplementary Figure 5. Mean fold changes in mRNA expression profile of virulence genes in spores (above) and spores + tissue (below). The data above show the normalized target gene amount in freshly collected spores of each isolate (n = 4) relative to freshly collected spores of *Bd*JEL423 (n = 4). The data below show the normalized target gene amount in spores that were incubated with skin tissue of midwife toads (n = 4) (*A. obstetricans*) for 2 h relative to freshly collected spores of the respective isolate (n = 4) which is considered 1. Boxes indicate 25th and 75th percentiles, central lines the median, bars the minima and maxima, and points indicate individual samples**.** Target genes were based on Farrer et al. (2017). An asterisk indicates a significant difference compared to *Bd*JEL423 spores (above: Kruskal–Wallis analysis, followed by pairwise Mann–Whitney U-tests (two-tailed) with a Benjamini-Hochberg adjusted *P* value < 0.05) or spores of the respective isolate (below: Kruskal–Wallis analysis, followed by pairwise Mann–Whitney U-tests (two-tailed), with a *P* value < 0.05). Individual *P* values are shown in Supplementary Tables 6-7. Source data are provided as a Source Data file.

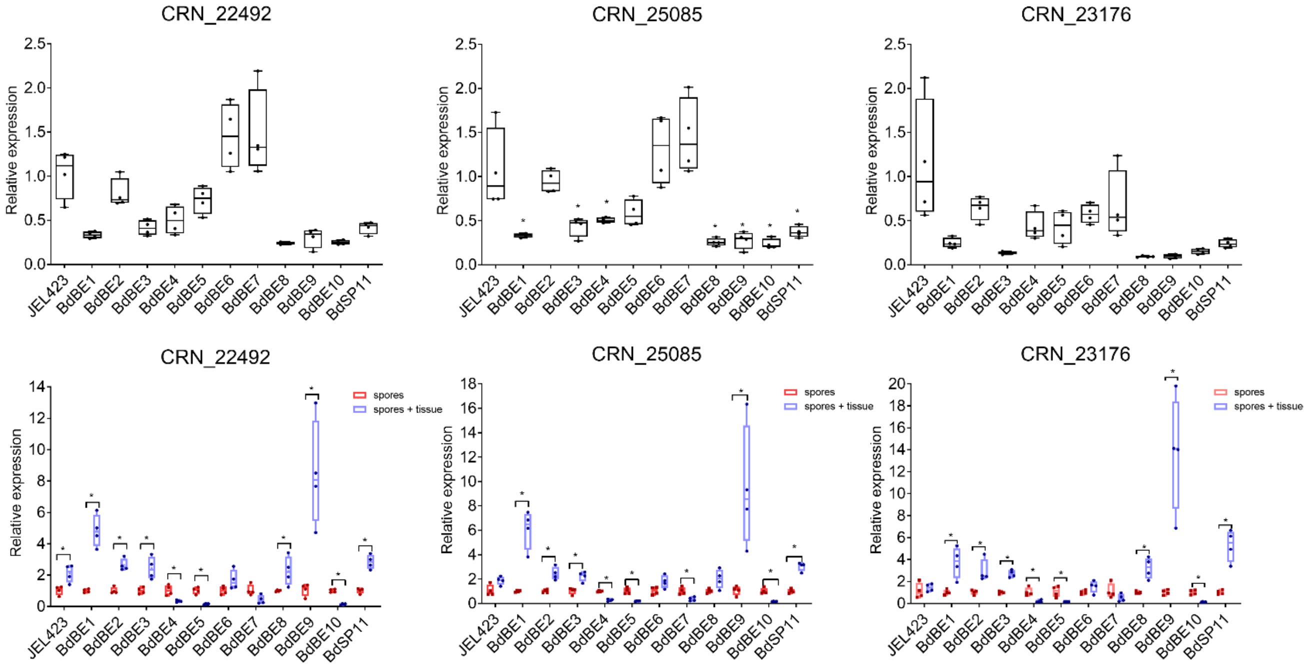

Supplementary Figure 6. Germ tube formation of different *B. dendrobatidis* strains. The individual fluorescent signals of *Bd*BE1, *Bd*BE3, *Bd*BE4, *Bd*BE5 and *Bd*JEL423 (Calcofluor White (blue)) and A6 cells (green cell tracker), were merged to assess the ability of germ tube (GT) formation of the different *Bd* strains, 4 hours after contact with the cells. Scale bar = 20 µm. To assess the *in vitro* infection dynamics of different *Bd*GPL isolates, three independent *in vitro* experiments were conducted with every condition being tested in triplicate, with similar results.

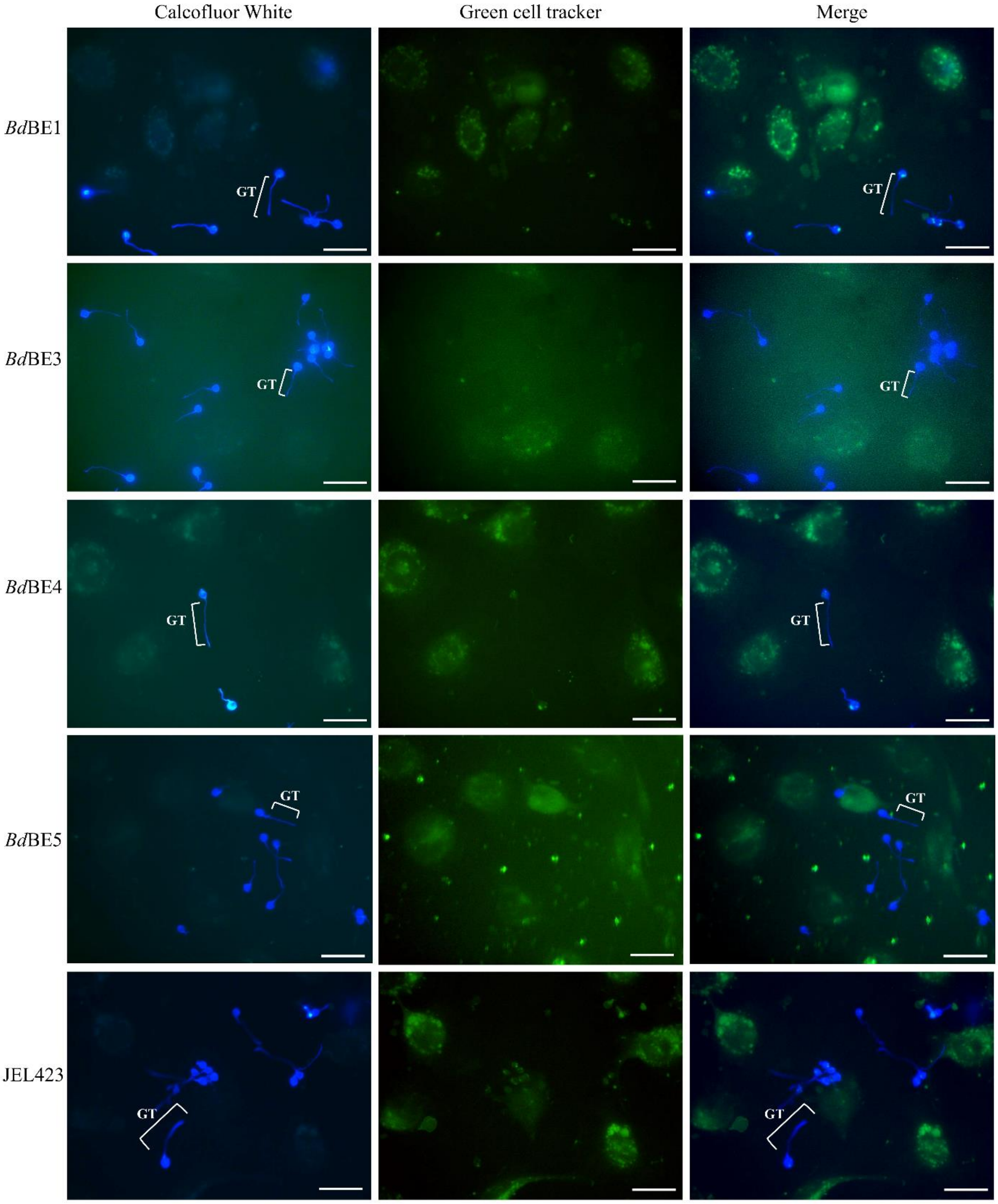

Supplementary Figure 7. Fluorescent stainings of the invasion capacity of different *B. dendrabotidis* strains. Two days post infection, the invasion capacity of *Bd*BE1, *Bd*BE3, *Bd*BE4, *Bd*BE5 and *Bd*JEL423 in A6 cells was assessed. *Bd*-exposed A6 cells were stained using a green cell tracker. *Bd* was visualized using Alexa Fluor 568 targeting a polyclonal antibody against *Bd* (Thomas et al., 2018), resulting in red fluorescence of both intracellular and extracellular *Bd*. The cell wall of extracellular *Bd* was coloured using Calcofluor White, showing blue fluorescence. * = intracellular colonization. Scale bar = 20 µm. To assess the *in vitro* infection dynamics of different *Bd*GPL isolates, three independent *in vitro* experiments were conducted with every condition being tested in triplicate, with similar results.

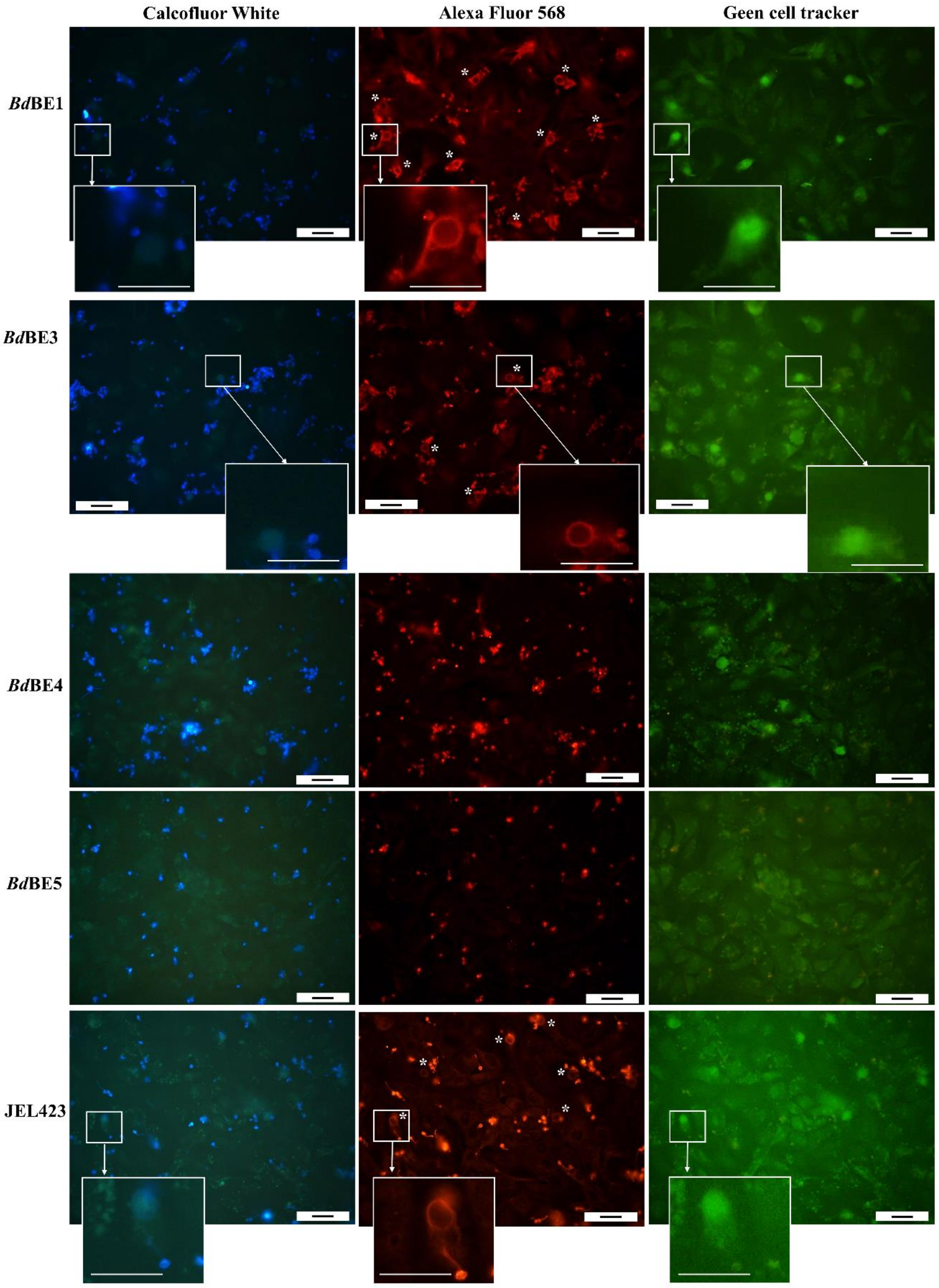

Supplementary Figure 8. Merged fluorescent stainings of the invasion capacity of different *B. dendrobatidis* strains. The fluorescent signals described in Supplementary Fig. 7 were merged. *Bd*BE1, *Bd*BE3 and *Bd*JEL423 show intracellular colonization (*), reflecting endobiotic growth where new intracellular chytrid thalli are formed by transfer of the cell content of the mother thallus through a germ tube to a new daughter thallus. Intracellular colonization was absent for the strains *Bd*BE4 and *Bd*BE5, which showed an epibiotic growth, limited to *Bd* development outside the host cells. GT= germ tube; MT = mother thallus; DT = daughter thallus; scale bar = 20 µm. To assess the *in vitro* infection dynamics of different *Bd*GPL isolates, three independent *in vitro* experiments were conducted with every condition being tested in triplicate, with similar results.

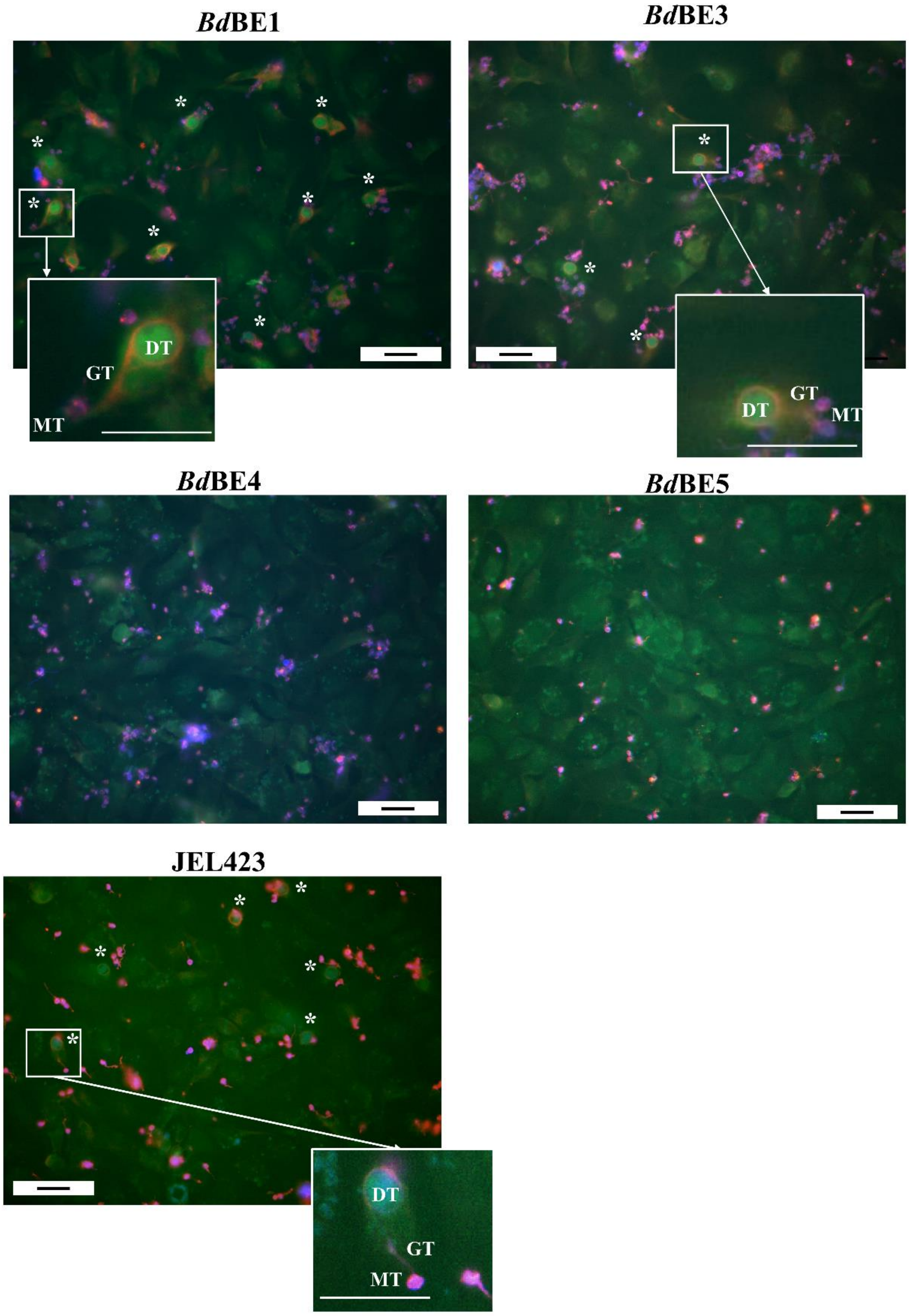

Supplementary Figure 9. Heat map depicting copy number variation of local *Bd* strains compared to *Bd*JEL423 in the 18 largest supercontigs. As a diploid organism, a copy number ratio log2 of ~0,585 represents addition of a single copy (log2(3/2)). Constructed using CNVkit heatmap command with –d flag to de-emphasise those low amplitude segments which are unlikely to represent true copy number variation.

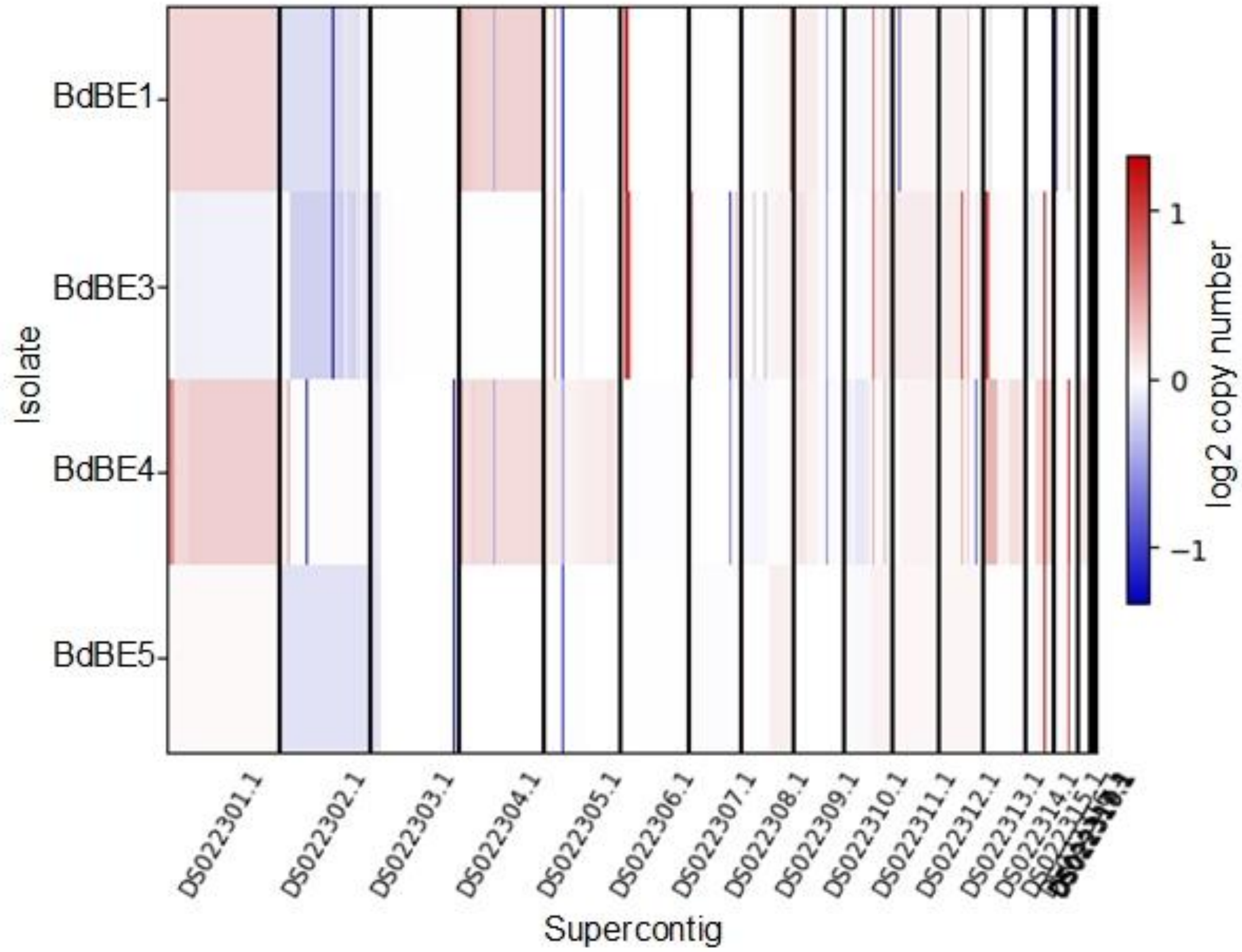

Supplement: Supplementary file 1 — Supplementary Information [file 41467_2020_19241_MOESM1_ESM.pdf]
