# Supplementary material for: Presence of low virulence chytrid fungi could protect European amphibians from more deadly strains
Source: Nat Commun. 2020 Oct 26;11:5393. doi: 10.1038/s41467-020-19241-7 (PMC7589487; doi:10.1038/s41467-020-19241-7)
Supplement: Supplementary file 3 — Reporting Summary [file 41467_2020_19241_MOESM3_ESM.pdf]

nature research

Corresponding author(s): An Martel

Last updated by author(s): Oct 2, 2020

Reporting Summary

Nature Research wishes to improve the reproducibility of the work that we publish. This form provides structure for consistency and transparency in reporting. For further information on Nature Research policies, see Authors & Referees and the Editorial Policy Checklist .

Statistics

For all statistical analyses, confirm that the following items are present in the figure legend, table legend, main text, or Methods section.

n/a	Confirmed	
☐	☒	The exact sample size (*n*) for each experimental group/condition, given as a discrete number and unit of measurement
☐	☒	A statement on whether measurements were taken from distinct samples or whether the same sample was measured repeatedly
☐	☒	The statistical test(s) used AND whether they are one- or two-sided<br>*Only common tests should be described solely by name; describe more complex techniques in the Methods section.*
☐	☒	A description of all covariates tested
☐	☒	A description of any assumptions or corrections, such as tests of normality and adjustment for multiple comparisons
☐	☒	A full description of the statistical parameters including central tendency (e.g. means) or other basic estimates (e.g. regression coefficient) AND variation (e.g. standard deviation) or associated estimates of uncertainty (e.g. confidence intervals)
☐	☒	For null hypothesis testing, the test statistic (e.g. *F*, *t*, *r*) with confidence intervals, effect sizes, degrees of freedom and *P* value noted<br>*Give P values as exact values whenever suitable.*
☒	☐	For Bayesian analysis, information on the choice of priors and Markov chain Monte Carlo settings
☒	☐	For hierarchical and complex designs, identification of the appropriate level for tests and full reporting of outcomes
☒	☐	Estimates of effect sizes (e.g. Cohen's *d*, Pearson's *r*), indicating how they were calculated

Our web collection on statistics for biologists contains articles on many of the points above.

Software and code

Policy information about availability of computer code

Data collection	No software was used to collect the data.
Data analysis	Image J 1.52d was used to analyse the phenotypic characteristics of the Bd isolates. QPCR results were analysed using the Bio-Rad CFX manager 3.1. Leica Application Suite (LAS) X software was used to analyse the in vitro infection dynamics of the Bd isolates.<br>For whole genome sequence analysis, we used BWA mem version 0.7.17, Picard tools version 2.21.1 AddOrReplace, MarkDuplicates, SortSam, CreateSequenceDictionary and ReorderSam, HaplotypeCaller GATK v4.1.4.0, CombineGVCFs, GenotypeGVCFs, SelectVariants, VariantFiltration, SNPeffv4.3, SignalP 4.0 server, TMHMM Server 2.0, HMMER 3.2.1 server, Blastp (BLAST+2.9.0) and CNVkit version 0.9.5.<br>Statistical anlysis was performed in R (package brglm2) or in SPSS (version 26).

For manuscripts utilizing custom algorithms or software that are central to the research but not yet described in published literature, software must be made available to editors/reviewers. We strongly encourage code deposition in a community repository (e.g. GitHub). See the Nature Research guidelines for submitting code & software for further information.

Data

Policy information about availability of data

All manuscripts must include a data availability statement. This statement should provide the following information, where applicable:

- Accession codes, unique identifiers, or web links for publicly available datasets
- A list of figures that have associated raw data
- A description of any restrictions on data availability

All data supporting this study are available in the article and corresponding supplementary information files. Source data for figures [1, 2, 3 and 4] and supplementary figures/Tables are provided in the source data file. Genomic read data are available at NCBI GenBank under Bioproject PRJNA413876 (SRA sample accession numbers: BdBE1; SRA: SRS2757215, BdBE3; SRA: SRS2757203, BdBE4; SRA: SRS2757202, BdBE5; SRA: SRS2757217, BdJEL423; SRA: SRS2757141). Reads were aligned to the BdJEL423 assembly available under BioProject PRJNA13653, assembly accession GCA_000149865.1.

Field-specific reporting

Please select the one below that is the best fit for your research. If you are not sure, read the appropriate sections before making your selection.

☐ Life sciences ☐ Behavioural & social sciences ☒ Ecological, evolutionary & environmental sciences

For a reference copy of the document with all sections, see nature.com/documents/nr-reporting-summary-flat.pdf

Ecological, evolutionary & environmental sciences study design

All studies must disclose on these points even when the disclosure is negative.

Study description

In this study we analysed the hypothesis that a co-existence of BdGPL with European amphibian communities is mediated by low virulence of local BdGPL lineages. We also explored the extent to which these low virulent isolates confer protection against invasion with highly virulent Bd or Bsal.

Therefore we:

1) Determined the occurrence and impact of local BdGPL in native amphibian populations in northern Europe.

• Opportunistic sampling of 1483 amphibians belonging to 62 populations (Flanders: 2015-2016). Sampling sites selected based on available data of amphibian breeding ponds in Flanders and accessibility.

• Targeted sampling of 5 breeding sites (Flanders) of midwife toads for 4 consecutive years (2015-2018), sites comprise all signficant breeding sites of the species in Flanders.

• Targeted sampling of 26 ponds (Flanders) for alpine newts, three or four times with one month interval (March-June 2019). Ponds were selected on prior, extensive sampling to determine amphibian community composition. The inclusion criterion was the presence of a reproducing population of alpine newts.

2) We compared virulence traits of the local BdGPL isolates (BdBE1-10) to those of the known hypervirulent BdGPL isolate JEL423.

• Phenotypic characterization of the different local Bd strains compared to JEL423, including number of zoospores (n = 4 independent experiments), number of sporangia (n = 4 independent experiments), area of the largest 10 sporangia (n = 10 technical replicates per independent experiment) and area of 10 random spores (n = 4 independent experiments)

• Susceptibility of native amphibians to BdGPL isolates: Infection dynamics of BdBE1 and JEL423 in seven amphibian species (n = 5) were analyzed: fire salamander (Salamandra salamandra), common spade foot (Pelobates fuscus), natterjack toad (Epidalea calamita), common toad (Bufo bufo), alpine newt (Icthyosaura alpestris), great crested newt (Triturus cristatus) and European tree frog (Hyla arborea))

• The growth ability of BdBE1 and JEL234 in mucosomes of midwife toads (Alytes obstetricans) and fire salamanders (Salamandra salamandra) was determined. The experiment was performed with 3 technical replicates and 10 biological replicates.

• BDGPL virulence in midwife toads: Four local phenotypically and genotypically1 different isolates originating from different species (BdBE1, BdBE3, BdBE4, and BdBE5) and the hypervirulent isolate BdJEL423 were selected to inoculate midwife toads (A. obstetricans). Thirty-six newly metamorphosed A. obstetricans were randomly assigned to groups of six for each treatment. Individuals were exposed to fresh spores, or distilled water and infection dynamics were analyzed.

• The mRNA expression of different virulence genes was analyzed in the local low virulent BdGPL isolates and the hypervirulent JEL423 strain whether or not exposed to skin tissue of Alytes obstetricans (n = 4 per condition)

• The in vitro adhesion and invasion capacity of BdBE1, BdBE3, BdBE4, BdBE5 and JEL423 was examined using fluorescent stainings. Three independent in vitro experiments were conducted with every condition being tested in triplicate, with similar results.

• Whole genome sequence data were mapped against the reference genome for BdJEL423 to identify genome-wide variation associated with the low virulence (BdBE1, BdBE3, BdBE4 and BdBE5) and cell invasion (BdBE1, BdBE3 and BdJEL423) phenotypes.

3) We quantified the protective capacity of low virulent BdGPL isolates against the hypervirulent BdGPL and Bsal.

• The remaining midwife toads from the BdGPL virulence trial were re-infected with a hypervirulent BdGPL (JEL423) and the protective capacity of the endemic BdGPL isolates was examined.

• The protective capacity of low virulent BdGPL against highly virulent Bsal was analyzed in three urodele species: fire salamanders (n = 27), ribbed newts (Pleurodeles waltl) (n = 24) and marbled newts (Triturus marmoratus) (n = 20) that were divided ad random in three treatment groups (every animal was housed individually). Two groups of each species were exposed to local BdGPL, three weeks later the one of these groups to Bsal. At this 21 day time point, a Bsal control group of each species was exposed to Bsal. Infection dynamics were analyzed.

• The protective capacity of the low virulent BdBE3 strain was confirmed in a second in vivo experiment. 40 newly metamorphosed A. obstetricans were randomly assigned to two groups and exposed to BdBE3, or distilled water. Animals were then housed individually and four weeks later, all animals were exposed to BdJEL423. Infection dynamics were analyzed.

Research sample

Batrachochytrium dendrobatidis isolates were obtained from wild amphibians isolated in 2015-2018.

BdBE1: Alytes obstetricans (Adult) -> 2015 (Huldenberg, Flemish-Brabant, Belgium)

BdBE2: Lithobates catesbeianus (Larvae) -> 2015 (Balen, Antwerp, Belgium)

BdBE3: Ichtyosaura alpestris (Adult) -> 2015 (Tubize, Walloon Brabant, Belgium)

BdBE4: Lithobates catesbeianus (Larvae) -> 2016 (Arendonk, Antwerp, Belgium)

BdBE5: Alytes obstetricans (Larvae) -> 2016 (Voeren, Limburg, Belgium)

BdBE6: Ichtyosaura alpestris (Adult) -> 2016 (Voeren, Limburg, Belgium)

BdBE7: Lithobates catesbeianus (Adult) -> 2016 (Laakdal, Antwerp, Belgium)

BdBE8: Lithobates catesbeianus (Adult) -> 2016 (Balen, Antwerp, Belgium)

BdBE9: Lithobates catesbeianus (Larvae) -> 2016 (Laakdal, Antwerp, Belgium)

BdBE10: Lithobates catesbeianus (Adult) -> 2016 (Balen, Antwerp, Belgium)
BdSP11: Ichtyosaura alpestris (Adult) -> 2018 (El Montnegr I El Corredor, Catalonia, Spain)

Infection dynamics were analyzed in different amphibian species comprising:
Fire salamanders (Salamandra salamandra), common spade foot (Pelobates fuscus), natterjack toad (Epidalea calamita), common toad (Bufo bufo), alpine newt (Ichtyosaura alpestris), great crested newt (Triturus cristatus), European tree frog (Hyla arborea), midwife toads (Altyes obstetricans), ribbed newts (Pleurodeles waltl) and marbled newts (Triturus marmoratus).

Genomic data: Genomic read data are available at NCBI GenBank under Bioproject PRJNA413876 (SRA sample accession numbers: BdBE1; SRA: SRS2757215, BdBE3; SRA: SRS2757203, BdBE4; SRA: SRS2757202, BdBE5; SRA: SRS2757217, BdJEL423; SRA: SRS2757141). Reads were aligned to the BdJEL423 assembly available under BioProject PRJNA13653, assembly accession GCA_000149865.1.

Sampling strategy

The sample size was based on previous experimental work with amphibians and Batrachochytrium dendrobatidis. Alytes obstetricans is the Bd sentinel anuran species, I. alpestris is considered a urodelan Bd supershedder and L. catesbeianus is an invasive alien species in Belgium. We anticipated that obtaining isolates from these species would result in maximum Bd diversity.

We sampled 1,483 amphibians belonging to 62 populations in 2015-2016. To detect the presence of Bd, we collected swabs from the superficial skin surface of metamorphosed animals or the mouthparts of larval anurans. To study potential co-existence of Bd in the study region with small populations of a susceptible species (where negative effects are expected to be most obvious), in a second study, we sampled five breeding sites of midwife toads in Flanders for four consecutive years. In these breeding sites, larvae were counted once a year and their mouthparts were sampled for the presence of Bd. In a third field study, we selected 26 ponds across our study area containing at least a population of alpine newt (Ichthyosaura alpestris), being the European urodele most likely infected by Bd. Ponds were sampled with funnel traps three or four times (depending on the presence of water) with a one month interval (March- June 2019). An envisaged 30 newts per sampling per pond were swabbed for the presence of Bd , weighed to the nearest 0.1g and the snout-vent length measured to the nearest mm to calculate SMI.

Animals were handled while wearing a fresh pair of non-powdered, disposable gloves. Equipment and field clothing were cleaned and disinfected between visits to sampling locations.

Detection of chytrid associated disease in the study region was done by post-mortem examination of field cases of amphibian disease or mortality over a period of four years. Wildlife Health Ghent hosts an amphibian disease hotline where suspect cases of infectious disease are submitted (cases with obvious traumatic causes such as predation or traffic are not withheld). The dead amphibians are routinely examined for the presence of Bd, Bsal and Ranavirus using (q)PCR.

Data collection

- Infection dynamics data were collected by sampling the skin using cotton-tipped swabs on which a DNA-extraction was performed, followed by qPCR (absolute: CFX384 RT-PCR cycler (Bio-Rad) and Bio-Rad CFX manager 3.1) to determine the Bd/Bsal loads. -> collected by Wouter Beukema, Mark Blooi, Robby Van Leeuwenberg, Mark Greener and An Martel
- Phenotypic characterization of the Bd isolates was performed using microscopy (Olympus CKX41 with an attached camera (Olympus SC50, Olympus)) in combination with image software analysis (ImageJ 1.52d software) -> collected by Mark Greener
- mRNA expression data were obtained via relative gene expression analysis. (absolute: CFX384 RT-PCR cycler (Bio-Rad) and Bio-Rad CFX manager 3.1)-> collected by Elin Verbrugghe
- In vitro growth test data were obtained after DNA extraction and qPCR (absolute: CFX384 RT-PCR cycler (Bio-Rad) and Bio-Rad CFX manager 3.1) > collected by Zhimin Li
- In vitro infection dynamics data were obtained via fluorescent microscopy and analysis (Leica Application Suite (LAS) software X) of the fluorescent images (ImageJ 1.52d software). -> collected by Elin Verbrugghe
- Whole genome sequence data were obtained from the NCBI bioproject PRJNA413876 (SRA sample accession numbers: (SRA sample accession numbers: BdBE1; SRA: SRS2757215, BdBE3; SRA: SRS2757203, BdBE4; SRA: SRS2757202, BdBE5; SRA: SRS2757217, BdJEL423; SRA: SRS2757141). -> Collected by Moira Kelly

Timing and spatial scale

Field work:
• Opportunistic sampling of 1483 amphibians belonging to 62 populations (Flanders) was done between 2/06/2015 and 17/11/2016.
• Sampling of 5 breeding sites (Flanders) of midwife toads was done for 4 consecutive years (2015-2018)
• Sampling of 26 ponds (Flanders) for alpine newts was done three or four times (depending on the presence of water) with one month interval (March-June 2019)

In vivo infection experiments:
• Susceptibility of native amphibians to BdGPL: Species that did not show infection for at least 3 consecutive weeks in any individual were removed from the experiment at week 8. For the species that became infected, the infection was followed up to 91 days post infection. The infection loads were followed up weekly.
• BdGPL isolate virulence for midwife toads: The infection dynamics were followed up to 91 days post infection. The infection loads were followed up weekly.
• Protective capacity of low virulent BdGPL isolates:
- After completion of the multi-isolate A. obstetricans infection trial, all remaining individuals were re-infected with BdJEL423. The infection dynamics were followed up to 12 weeks post infection. The infection loads were followed up weekly.
- The protective capacity of the low virulent BdBE3 strain was confirmed in an independent experiment where newly

	metamorphosed A. obstetricans were first exposed to the low virulent BdBE3 and 4 weeks later to the hypervirulent JEL423.<br>- The protective capacity of low virulent BdGPL against highly virulent Bsal was examined in three urodele species, up to 32 weeks post infection. The infection loads were followed up weekly.
Data exclusions	No data were excluded from the analysis.
Reproducibility	Field work:<br>A large amount of animals was sampled and over a large time scale (2015, 2016, 2017, 2018 and 2019). All data show a stable endemism of BdGPL with a minor cost for host populations.<br><br>In vivo infection experiments:<br>• To maximize reproducibility, all amphibians used in experimental trials were captive bred and had no known history of Bd infection (which may interfere with infection dynamics).<br>Susceptibility of native amphibians to BdGPL: This experiment highlights the marked susceptibility of midwife toads to BdGPL and the low susceptibility of common toads and fire salamanders. This was reproduced using an ex vivo growth experiment with mucosomes collected from midwife toads, common toads and fire salamanders. For each species, the mucosomes were collected from three different animals and they all confirm the in vivo results.<br>The susceptibility of midwife toads to BdGPLwas also reproduced in a second in vivo trial that analyzed the BdGPL isolate virulence for midwife toads. This experiment showed that midwife toads are highly susceptible to exotic, virulent BdGPL(JEL423), but that they tolerate infection with endemic BdGPL.<br>• Protective capacity of low virulent BdGPL: In three independent in vivo trials, using different amphibian species, the protective capacity of endemic BdGPL against virulent BdGPL/Bsal was shown.<br><br>In vitro/ex vivo experiments:<br>• Phenotypic characterization of Belgian Bd isolates: The experiment was performed in quadruplicate to increase the reproducibility.<br>• BdGPL gene expression: All conditions were analyzed in fourfold (biological replicates) and QPCR reactions were run in triplicate (technical replicates).<br>• Mucosome experiments: All conditions were analyzed in tenfold (biological replicates) and were run in triplicate (technical replicates).<br>• In vitro infection of A6 cells: The experiment was conducted in triplicate and was repeated 3 times independently.<br>All attempts at replication of in vitro/ex vivo experiments were successful.
Randomization	In vivo infection experiments: all individuals were randomly assigned to treatments.<br>In vitro/ex vivo experiments:<br>• Phenotypic characterization of Belgian Bd isolates: This could not be randomized because different isolates were assessed.<br>• BdGPL gene expression: This could not be randomized because different isolates were assessed.<br>• Mucosome experiments: This could not be randomized because different isolates were assessed.<br>• In vitro infection of A6 cells: This could not be randomized because different isolates were assessed.
Blinding	In vivo infection experiments: The investigators were not blinded during data collection to minimize contamination. All in vivo sample analysis was blinded.<br>In vitro/ex vivo experiments: The investigators were not blinded to group allocation during data collection or analysis since different conditions needed to be tested and control conditions are always included as a reference during the experimental analysis to consolidate the experimental procedures.

Did the study involve field work? ☒ Yes ☐ No

Field work, collection and transport

Field conditions	• Opportunistic sampling of 1483 amphibians belonging to 62 populations was done in 2015-2016.<br>• Sampling of 5 breeding sites (Flanders) of midwife toads was done for 4 consecutive years (2015-2018)<br>• Sampling of 26 ponds (Flanders) for alpine newts with funnel traps was done three or four times (depending on the presence of water) with one month interval (March-June 2019)<br><br>Field work was performed during activity periods of the amphibian target species.
Location	Variable, due to our sampling campaign targeting terrestrial, aquatic and semi-aquatic amphibians across the Belgian region of Flanders.<br>• Opportunistic sampling of amphibians: This was done in Flanders, with positive animals found in (Lat, Lon): Raffelberg (50,822931 4,594920), Straalmolen (51,143872 5,128007), Hoogstraten (51,424806 4,821099), Zandgroeve Neerijse (50,819035 4,607852), Zonhoven (50,995471 5,316816), Heilig Geestgoed (50,940000 3,730000), Duffel (51,081411 4,501465), Veerle (51,075030 4,967758), Olmen (51,146314 5,142116), Groot-Loon (50,793582 5,361510), 's Gravensvoeren (50,757401 5,800900), Hasselt (50,932999 5,310000), Arendonk (51,325001 5,113000), Zandgrove Neerijse (50,819000 4,607000), Arendonk (51,325001 5,113000) and Neerijse (50,822899 4,594900).

	• Sampling of 5 breeding sites: This was done in Neerijse, Rijkel, Groot-Loon, 's Gravensvoeren, Sint-Genesius-Rode<br><br>• Sampling of 26 ponds: This was done in Brakel, Lierde, Zottegem and Maarkedal
Access and import/export	Field surveys and amphibian collection were arranged through permits ANB/BL/FF-V15-00131 and ANB/BL/FF-V1800013 from the Agentschap voor Natuur en Bos (ANB), Belgium. We arranged access to amphibian habitats located on private land (i.e. not owned by ANB) prior to visiting.
Disturbance	Disturbance was minimized by using fikes to catch newts, instead of dip netting, and looking for terrestrial amphibians at night during rain instead of actively searching by lifting logs, rocks etc.

Reporting for specific materials, systems and methods

We require information from authors about some types of materials, experimental systems and methods used in many studies. Here, indicate whether each material, system or method listed is relevant to your study. If you are not sure if a list item applies to your research, read the appropriate section before selecting a response.

Materials & experimental systems

n/a	Involved in the study	
☐	☒	Antibodies
☐	☒	Eukaryotic cell lines
☒	☐	Palaeontology
☐	☒	Animals and other organisms
☒	☐	Human research participants
☒	☐	Clinical data

Methods

n/a	Involved in the study	
☒	☐	ChIP-seq
☒	☐	Flow cytometry
☒	☐	MRI-based neuroimaging

Antibodies

Antibodies used	1) polyclonal antibody against Bd: This antibody was raised in rabbits during an immunisation trial, as described in Thomas et al. (2018).<br>Thomas, V. et al. Recommendations on diagnostic tools for Batrachochytrium salamandrivorans. Transbound Emerg Dis 65, e478–e488 (2018).<br><br>2) goat anti-rabbit Alexa Fluor 568: Fisher Scientific; A11011
Validation	Validation of the antibodies was performed previously in Verbrugghe et al. (2019).<br>Verbrugghe, E., Van Rooij, P., Favoreel, H., Martel, A., Pasmans F. (2019) In vitro modeling of Batrachochytrium dendrobatidis infection of the amphibian skin. PLos One 14: e0225224

Eukaryotic cell lines

Policy information about cell lines

Cell line source(s)	Xenopus laevis kidney epithelial cell line A6 which was obtained from ATCC (ATCC-CCL 102)<br>https://www.lgcstandards-atcc.org/Products/All/CCL-102.aspx?geo_country=be#
Authentication	The is a commercially available continuous cell line that was obtained from ATCC (ATCC-CCL 102) validating the authentication with a certificate of Analysis. Therefore no additional authentication procedures were performed.
Mycoplasma contamination	Cells were not tested for mycoplasma contamination.
Commonly misidentified lines<br>(See ICLAC register)	No commonly misidentified cell lines were used in the study.

Animals and other organisms

Policy information about studies involving animals; ARRIVE guidelines recommended for reporting animal research

Laboratory animals	Animals (males/females) were randomly assigned to treatments. All animals used had no prior history of Bd infection (sampled at least twice before the onset of the experiment).<br>Animals included were:<br>• Susceptibility of native amphibians to BdGPL: fire salamander (Salamandra salamandra), common spade foot (Pelobates fuscus), natterjack toad (Epidalea calamita), common toad (Bufo bufo), alpine newt (Icthyosaura alpestris), great crested newt

	(Triturus cristatus), European tree frog (Hyla arborea)), midwife toads (Alytes obstetricans)<br>• BdGPL virulence for midwife toads: newly metamorphosed midwife toads (Alytes obstetricans)<br>• protective capacity of low virulent BdGPL: midwife toads (Alytes obstetricans; remaining individuals from the BdGPL virulance infection trial), fire salamander (Salamandra salamandra), ribbed newts (Pleurodeles waltl) and marbled newts (Triturus marmoratus).
Wild animals	Field animals were sampled by (non invasive) swabbing their skin. Alpine newts that were sampled in March-June 2019 were also weighed and the snout-vent length was measured. No animals were transported.
Field-collected samples	The skin swabs were stored at -20°C until further analysis.
Ethics oversight	Field surveys and amphibian collection were arranged through permits ANB/BL/FF-V15-00131 and ANB/BL/FF-V1800013 from the Agentschap voor Natuur en Bos (ANB), Belgium. All infection trials were approved by the Ethical Committee of the Faculty of Veterinary Medicine, UGent.

Note that full information on the approval of the study protocol must also be provided in the manuscript.
